# Cholesterol Trafficking: An Emerging Therapeutic Target for Angiogenesis and Cancer

**DOI:** 10.3390/cells8050389

**Published:** 2019-04-28

**Authors:** Junfang Lyu, Eun Ju Yang, Joong Sup Shim

**Affiliations:** Cancer Centre, Faculty of Health Sciences, University of Macau, Taipa, Macau 999078, China; junfanglyu@um.edu.mo (J.L.); ejyang@um.edu.mo (E.J.Y.)

**Keywords:** cholesterol trafficking, angiogenesis, lysosome, NPC1, mTOR

## Abstract

Cholesterol is an essential structural component of cellular membranes. In addition to the structural role, it also serves as a precursor to a variety of steroid hormones and has diverse functions in intracellular signal transduction. As one of its functions in cell signaling, recent evidence suggests that cholesterol plays a key role in regulating angiogenesis. This review discusses the role of cholesterol in angiogenesis, with a particular emphasis on cholesterol trafficking in endothelial cell signaling. Small molecule inhibitors of cholesterol trafficking and their preclinical and clinical development targeting angiogenesis and cancer are also discussed.

## 1. Cholesterol Synthesis and Distribution

Cholesterol is an indispensable constituent of cellular membranes and plays a critical role in membrane permeability and fluidity. In addition to structural support, it also functions in intracellular transport and cell signaling as a critical component of lipid rafts [1,2]. Understanding cholesterol synthesis, cellular uptake, and trafficking is important because the proper distribution of cholesterol in the organelles is critical for cellular functions [3,4,5]. There are two sources of cholesterol, that which is synthesized in the endoplasmic reticulum (ER) [6], and that which is absorbed from the extracellular space via low-density lipoprotein (LDL) receptor-mediated endocytosis [7]. Cholesterol is synthesized in the ER from acetate in a complex process involving over 30 enzymatic steps, including the conversion of acetyl-CoA to 3-hydroxy-3-methylglutaryl-CoA (HMG-CoA) by HMG-CoA synthase, and the irreversible conversion to mevalonate by the rate-limiting enzyme HMG-CoA reductase (HMGCR). Newly synthesized cholesterol from the ER is transported to the plasma membrane, either directly or via the Golgi [8]. Dietary cholesterol is absorbed from the gastrointestinal tract, where cholesterol and triglycerides are packaged to form chylomicrons. Chylomicrons are modified in the circulation to form chylomicron remnants that are then transported to the liver [8]. In the liver, hepatocytes secrete lipids and cholesterol in very low-density lipoprotein (VLDL) particles that are further modified to LDL in the circulation before being delivered to peripheral cells. Excess cholesterol from the peripheral cells is released to high-density lipoproteins (HDL) that return the lipids and cholesterol to the liver through a process called reverse cholesterol transport [9].

Cholesterol homeostasis is tightly modulated by a complex network which involves its synthesis, import, export, esterification, and metabolism [8]. In the ER membrane, sterol regulatory element-binding proteins (SREBP), especially SREBP2 and 1a, are critical regulators of the genes involved in cholesterol uptake and biosynthesis, such as LDL receptors and HMGCR [10]. ER cholesterol acts as a sensor of intracellular cholesterol. The decrease in ER cholesterol induces the translocation of SREBP from the ER to the Golgi, and mature SREBP is transported into the nucleus for the transcriptional activation of the target genes, including those involved in cholesterol uptake and biosynthesis [8]. Increased intracellular cholesterol levels turn off cholesterol synthesis by trapping SREBP in the ER membrane via a sterol-mediated, protein–protein interaction with SCAP (SREBP cleavage-activating protein) and INSIG-1 [11]. Excess cholesterol is removed by an HDL-mediated efflux of cholesterol [12]. The liver X receptors (LXR) regulate the expression of genes involved in the cholesterol efflux, such as the adenosine triphosphate-binding cassette (ABC) transporters ABCA1 and ABCG1 [13].

**Figure 1:**
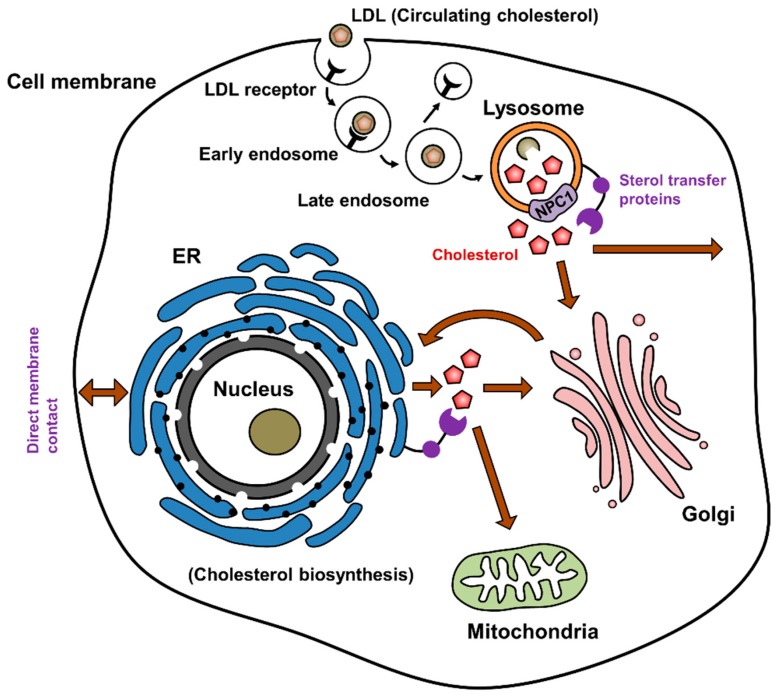
Intracellular cholesterol trafficking. Biosynthesized cholesterol from endoplasmic reticulum (ER) is delivered to the cell membrane directly or via the Golgi. Circulating cholesterol (low-density lipoprotein (LDL)-cholesterol) is delivered through the endosomal trafficking system, where LDL binds to the LDL receptor in the clathrin-coated pits on the cell membrane and internalizes via endocytosis. The endosomes are then mature and are fused with lysosomes, where cholesterol is released from LDL and transported out from the endosomal system. Through anterograde (ER to Golgi) and retrograde (Golgi to ER) trafficking, cholesterol is delivered to cellular organelles and plasma membrane. Inter-compartmental cholesterol delivery can be done via sterol transfer proteins, such as oxysterol binding protein (OSBP) and OSBP-related protein (ORP), or by membrane (vesicular) trafficking along cytoskeletal proteins using kinesin or dynein motors.

**Figure 2:**
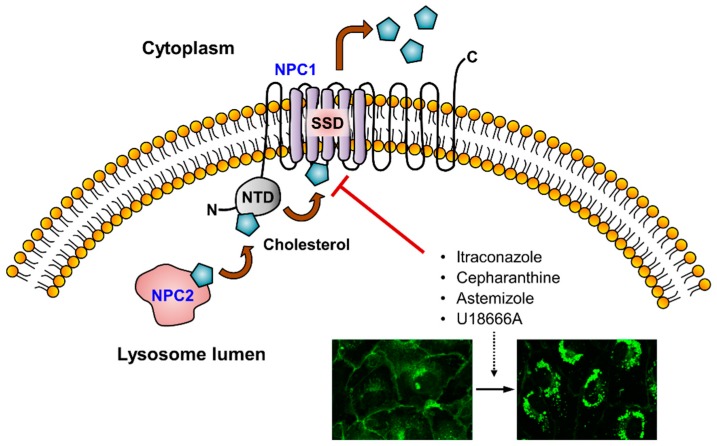
Cholesterol transport in the lysosome. Upon being delivered to the lysosome, LDL and cholesteryl esters are hydrolyzed by lysosomal acid lipase (LAL). Unesterified cholesterol in the lysosome lumen binds to Niemann-Pick disease type C (NPC) NPC2 and is then transferred to the N-terminal domain (NTD) of NPC1 in the inner-membrane side of the lysosome. Cholesterol is transferred from the NTD to the sterol-sensing domain (SSD) of NPC1 and transported out of the lysosome. Several cholesterol trafficking inhibitors including itraconazole, cepharanthine, and astemizole, as well as U18666A, are known to bind to the SSD and interfere with the cholesterol transport by NPC1. This effect leads to an accumulation of free cholesterol inside the lysosome. The immunofluorescence images are from endothelial cells stained with filipin (cholesterol-specific fluorescent dye). The left immunofluorescence image is control HUVEC with a high level of cholesterol in the cell membrane and other intracellular compartments. The right one is an example of HUVEC treated with cepharanthine, showing the strong accumulation of cholesterol in the lysosomes and the depletion of cholesterol in other cellular compartments.

Extracellular cholesterol (LDL) absorption and distribution into cells requires an appropriate endosomal trafficking system (Figure 1). LDL binds to its receptor and is then absorbed by clathrin-mediated endocytosis. Upon internalization, LDL is delivered to early sorting endosomes and then to late endolysosomes, where LDL and cholesteryl esters are hydrolyzed, after which the LDL receptor can be recycled back to the plasma membrane [8]. After hydrolyzing cholesteryl esters by lysosomal acid lipase (LAL), the Niemann-Pick type C (NPC) proteins (NPC1 and NPC2) are required for transporting free cholesterol out of the lysosome [14]. Mutations in NPC1 or 2 result in the accumulation of unesterified cholesterol and glycolipids in lysosomes causing an inherited lysosomal storage disease, called Niemann-Pick disease type C (NPC) [15]. NPC1 is a membrane protein comprising of 13 transmembrane helices and 3 luminal domains [16], while NPC2 is a soluble lysosomal luminal protein [17]. Based on the structural studies, it has been proposed that unesterified cholesterol binds to NPC2 in the lysosomal lumen and NPC2 transfers it to the N-terminal domain (NTD) of NPC1 on the inner-membrane side [14]. Cholesterol is then further transferred to the sterol-sensing domain (SSD) in the third transmembrane helix of NPC1, where cholesterol is finally transferred across the lysosomal membrane to exit from the lysosomes (Figure 2) [18]. Cholesterol is then delivered to other compartments, including the plasma membrane, the ER, and the mitochondria via membrane transport or by using sterol transfer proteins [8].

## 2. Cholesterol and Angiogenic Signaling

Extracellular cholesterol (circulating LDL) in the blood vessels needs to enter or pass through the endothelium layer to be distributed to the whole body. Therefore, blood vessels and endothelial/vascular smooth muscle cells play an important role in whole-body LDL penetration, accumulation, and metabolism [19]. Conversely, it has been increasingly suggested that cholesterol also plays an important role in endothelial cell functions and angiogenesis for its proper transport and distribution in the body. In this section, we introduce diverse evidence that reveals the potential roles of cholesterol in endothelial cell functions and angiogenesis.

### 2.1. Cholesterol Biosynthesis and Angiogenesis

In the late 1990s, several groups reported possible connections between cellular cholesterol levels and angiogenesis based on the observation that HMGCR inhibitors (statins) that inhibit cholesterol synthesis could modulate angiogenesis. Negre-Aminou et al. [20] reported that several HMGCR inhibitors inhibited human vascular smooth muscle cell (HSMC) proliferation. In particular, pravastatin showed a selective inhibition against HSMC over other types of cells, such as human cornea fibroblasts or human myoblasts. Feleszko et al. [21] showed that lovastatin significantly inhibited tumor angiogenesis by reducing VEGF level and potentiated the antitumor effects of TNF-α in a murine tumor model. Vincent et al. [22] reported that the HMGCR inhibitor cerivastatin inhibited endothelial cell migration to contribute to its anti-angiogenic effect. These reports suggested that the inhibition of cholesterol biosynthesis pathways could block angiogenesis. In contrast, another study showed an opposite effect of HMGCR inhibitors on angiogenesis, i.e., the HMGCR inhibitor simvastatin activated AKT and promoted angiogenesis in normocholesterolemic animals [23]. This observation was likely due to the biphasic effect of HMGCR inhibitors on angiogenesis. Weis et al. [24] reported that HMGCR inhibitors at low nanomolar concentrations enhanced endothelial cell proliferation, migration, and differentiation, but it significantly inhibited angiogenesis at high-nanomolar or single-digit micromolar concentrations. This biphasic effect was also observed in a murine tumor model where HMGCR inhibitors enhanced inflammation-induced angiogenesis at a low-dose, while they significantly inhibited at a high-dose therapy. Based on their in vitro and preclinical evidences of anti-angiogenic and antitumor effects, a number of HMGCR inhibitors are currently under clinical investigations in patients with various tumor types (https://clinicaltrials.gov). In addition to the cholesterol biosynthesis inhibitors, the body cholesterol absorption inhibitor ezetimibe has also been reported to inhibit angiogenesis. Ezetimibe (Zetia) is an FDA-approved, cholesterol uptake-blocking drug which works by binding to and inhibiting NPC1-like 1 (NPC1L1) protein, a gastrointestinal tract transporter responsible for dietary and biliary cholesterol absorption [25]. Solomon et al. [26] reported that a hypercholesterolemic diet elevated circulating cholesterol levels and promoted angiogenesis in vivo and the growth of prostate cancer xenograft tumors, compared to those observed in a hypocholesterolemic diet. Ezetimibe treatment significantly reduced circulating cholesterol levels and inhibited tumor angiogenesis in the mouse model. These data strongly suggest that cholesterol level is one of key factors regulating angiogenesis.

### 2.2. Endothelial Cell Cholesterol Level and Angiogenic Signaling

Cholesterol is an important component of lipid rafts in the cell membrane where the membrane proteins are anchored. Lipid rafts in the cell membrane function in cell signaling by serving as scaffold structures for signaling molecules [27]. The depletion of cholesterol in lipid rafts disrupts multiple signaling processes related to cell adhesion, migration, survival, and proliferation [27,28]. Several recent reports suggest that the membrane cholesterol content in endothelial cells determines angiogenic fate. Fang et al. [29] reported that apoA-I binding protein (AIBP)-mediated cholesterol efflux in endothelial cells is critical for proper angiogenesis. AIBP is a secreted protein that binds to HDL and facilitates cholesterol efflux from the cells to HDL through the ABCG1 transporter protein. Over-expression of AIBP led to the depletion of endothelial cell cholesterol and reduced lipid rafts on the cell membrane. This effect in turn interfered with VEGFR2 dimerization on the endothelial cell membrane and inhibited signaling and angiogenesis in vitro and in vivo. Similarly, Noghero et al. [30] reported that LXRs regulated angiogenesis via altering endothelial cell cholesterol levels. LXRs belong to the nuclear hormone receptor super-family that regulate cellular and systemic cholesterol homeostasis by reducing cholesterol absorption and inhibiting cholesterol biosynthesis [31]. Treatment of endothelial cells with LXR agonists reduced endothelial cell cholesterol levels, inhibited endothelial cell proliferation, migration, and tubulogenesis, and blocked in vivo neo-angiogenesis and tumor angiogenesis in mice models. These effects were significantly rescued by adding exogenous cholesterol, suggesting that cholesterol depletion was the primary mechanism of the anti-angiogenic effect of the LXR agonists. They further showed that cholesterol depletion which was mediated by LXR agonists reduced compartmentation of VEGFR2 in lipid raft/caveolae and inhibited VEFGR2 signaling in endothelial cells. These data further demonstrated that endothelial cell cholesterol levels are crucial for proper angiogenic signaling.

## 3. Cholesterol Trafficking Inhibitors as Anti-Angiogenic Agents

We discussed how endothelial cell cholesterol levels affect angiogenic signaling. Depletion of intracellular cholesterol in turn reduces the amount of plasma membrane cholesterol that affects lipid raft structure and interferes with the major angiogenic receptor signaling on the cell membrane. In addition to the total cholesterol level, intracellular cholesterol distribution governed by cholesterol trafficking is also essential for proper functioning of lipid rafts in the plasma membrane and other cellular membranous organelles. We, and other groups, have identified several small molecule inhibitors of endothelial cell cholesterol trafficking and proposed a potential role of cholesterol trafficking in angiogenesis based on their pharmacological effects.

### 3.1. Itraconazole

Itraconazole is an azole antifungal drug approved for patients with fungal infections. From a screening for anti-angiogenic agents using a clinical drug library, Chong et al. [32] identified itraconazole as a potential inhibitor of endothelial cell proliferation. In a series of follow-up animal and preclinical studies, itraconazole was found to be a potent inhibitor of angiogenesis and cancer [33,34,35,36]. In mechanistic studies, Xu et al. [37] first reported that itraconazole inhibited cholesterol trafficking and suppressed mTOR signaling in endothelial cells. Treatment of human umbilical vein endothelial cells (HUVEC) with itraconazole caused a high accumulation of free cholesterol in the late endolysosomes with little detected in the cell membrane and other cellular compartments, a phenotype similar to NPC. Replenishing endothelial cells with exogenous cholesterol significantly rescued the anti-proliferative effect of itraconazole, suggesting that cholesterol trafficking inhibition was likely the primary effect of itraconazole on endothelial cells. Recently, Head et al. [38] developed a photoaffinity probe of itraconazole and elaborated molecular targets and the mechanism of cholesterol trafficking inhibition in endothelial cells using the probe. Based on the competitive binding assays and structural modeling, itraconazole was found to directly bind to the SSD of human NPC1 and block cholesterol transport from the lysosomes, causing cholesterol accumulation inside the lumen (Figure 2). The role of cholesterol trafficking in mTOR signaling was verified by the rescue effect of exogenous cholesterol on itraconazole inhibition of mTOR signaling. However, the mechanism by which cholesterol trafficking inhibition influenced mTOR activity remained elusive. Nevertheless, itraconazole, as the most clinically advanced drug with a cholesterol trafficking inhibitory effect, has entered a number of clinical trials for patients with various types of cancer [39,40,41,42].

### 3.2. Selective Estrogen Receptor Modulators (SERM)

Selective estrogen receptor modulators (SERM), such as tamoxifen, are estrogen receptor antagonists that have long been used for the treatment of patients with estrogen receptor-positive breast cancer since the 1980s. However, several studies revealed that tamoxifen also showed an anticancer effect in estrogen receptor-negative breast cancer [43,44,45]. Later, researchers discovered that tamoxifen and other SERM were strong inhibitors of angiogenesis, independently of their inhibitory effect on estrogen receptors [46,47,48]. However, the molecular mechanism that allows SERM to inhibit angiogenesis has not been explained until recently. Our group recently found that SERM, including tamoxifen, toremifene, clomifene, and raloxifene, inhibited cholesterol trafficking in endothelial cells [49]. Treatment of HUVEC with SERM strongly induced the NPC-like phenotype in the cells and blocked VEGFR2 and mTOR signaling. We further verified that the inhibition of cholesterol trafficking by SERM was not due to their inhibitory effect on estrogen receptors. SERM’s inhibitory effects on VEGFR2 and mTOR signaling, as well as angiogenesis, were all significantly rescued by replenishing endothelial cells with cholesterol, suggesting that the inhibition of cholesterol trafficking was the primary effect of SERM for their anti-angiogenic activity. However, molecular targets, other than estrogen receptors, of tamoxifen responsible for the inhibition of cholesterol trafficking have not been elucidated. One possible explanation comes from the fact that tamoxifen is known to inhibit acidification of cellular acidic organelles, such as endosomes and lysosomes [50]. Treatment of drug-resistant cancer cells with tamoxifen significantly increased lysosomal pH, thereby redistributing weak-base chemotherapeutics sequestered in acidic organelles to their sites of action in the nucleus. This effect of tamoxifen sensitized drug-resistant cancer cells to DNA-targeting chemotherapeutic drugs. It has been proposed that tamoxifen as a tertiary amine can be protonated and trapped in acidic organelles and neutralize the organelles pH [51]. Therefore, it can be hypothesized that the increase in lysosomal pH by tamoxifen could block the functions of the lysosomal acidophilic enzymes and interfere with the endosomal cholesterol trafficking that requires a series of lysosomal enzymes.

### 3.3. Cepharanthine and Astemizole

Cepharanthine is an approved drug in Japan used for various illnesses such as inflammation and the alleviation of chemotherapy-induced adverse effects [52]. In clinical studies, cepharanthine showed several beneficial effects when combined with chemotherapy, i.e., potentiating the anti-tumor effect of chemotherapy and reducing the adverse effects associated with chemo-radio therapy [53,54,55]. However, the molecular mechanisms underlying its pharmacological activities remained elusive. From our imaging-based phenotypic screening using the cholesterol-binding fluorescent dye, filipin, cepharanthine was one of the strongest inhibitors of endothelial cell cholesterol trafficking [56]. Cepharanthine treatment induced a high accumulation of free cholesterol in the late endolysosomes, while it reduced cholesterol levels in other cellular compartments. Our in-depth mechanistic studies revealed two different target-points of cepharanthine for its cholesterol trafficking inhibitory effect, i.e., (1) It increases lysosomal pH, thereby interfering with the lysosomal enzymes responsible for LDL degradation and hydrolysis of cholesteryl esters, and (2) it directly binds to the SSD of NPC1, thereby blocking cholesterol transport from the lysosomes. Cepharanthine inhibited mTOR signaling in endothelial cells and strongly suppressed angiogenesis in vivo in a cholesterol-dependent manner, suggesting that the blockade of cholesterol trafficking was the primary effect for its anti-mTOR and anti-angiogenic activity. From the screening, we also identified the anti-histamine drug astemizole as a cholesterol trafficking inhibitor. Both cepharanthine and astemizole were shown to bind to the SSD of NPC1 in a ligand competition assay and molecular docking simulation [57]. From our pharmacological studies with the two drugs, we found that the cepharanthine and astemizole commonly caused the accumulation of cholesterol in the late endolysosomes, leading to the depletion of intracellular cholesterol, especially in membranous structures. This effect in turn dissociated mTOR from the lysosomes, causing its inactivation. Replenishing endothelial cells with cholesterol induced mTOR association back to the lysosomal surface, and rescued mTOR inhibition by cepharanthine or astemizole. Intracellular cholesterol depletion by cepharanthine or astemizole was confirmed by SREBP nuclear translocation after being treated with cepharanthine or astemizole and its reversal by cholesterol replenishment. These data demonstrated that blocking cholesterol trafficking leads to the accumulation of cholesterol in a compartment where the blockade occurs, causing its depletion in several intracellular membrane compartments where cholesterol is essential for the function of angiogenic signaling proteins, such as mTOR and VEGFR2.

## 4. Summary and Future Perspectives

**Figure 3:**
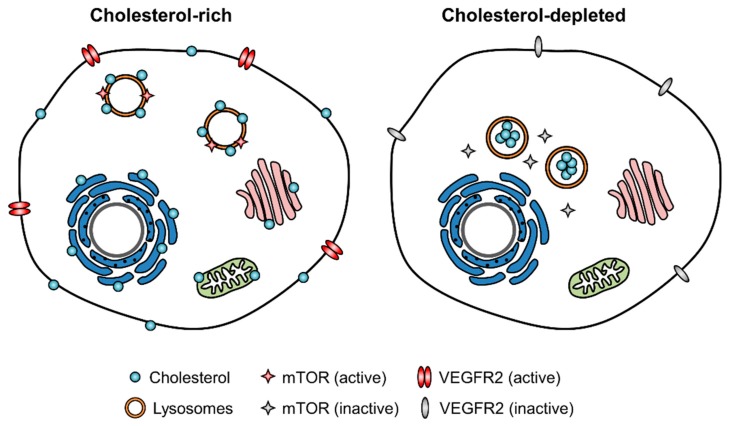
Endothelial cell cholesterol level and angiogenic signaling molecules. The proper level of cholesterol in the membrane is critical for membrane structure and the function of membrane binding angiogenic signaling proteins, such as mTOR and VEGFR2. Depletion of cholesterol by the inhibition of cholesterol trafficking leads to the dissociation of mTOR from the lysosomal membrane and the inhibition of VEGFR2 dimerization on the cell membrane, causing the inhibition of their signaling pathways.

In this review, we overviewed cholesterol synthesis, regulation, and distribution, and discussed the potential role of cholesterol levels and trafficking in angiogenesis based on experimental evidence. There are several cholesterol sensing mechanisms in cells, such as SCAP-INSIG-1-SREBP and squalene monooxygenase, to maintain proper cholesterol levels [8,10,11,58]. Maintaining proper levels of intracellular cholesterol is very important as the level regulates the functions of several key membrane signaling proteins. In endothelial cells, VEGFR2 and mTOR are major signaling proteins that are regulated by cholesterol levels on the cell membrane and lysosomal membrane, respectively [29,37,49,56,57]. Body or intracellular cholesterol levels can be modulated by changing cholesterol biosynthesis, cholesterol absorption from the gastrointestinal tract, cellular cholesterol efflux, and intracellular cholesterol trafficking. Intracellular cholesterol trafficking is as important as cholesterol biosynthesis because proper trafficking is essential for the delivery of synthesized cholesterol to the place where it is necessary. We further introduced several pharmacological inhibitors of cholesterol trafficking that were recently identified from the clinical drug screenings. From the pharmacological studies of the cholesterol trafficking inhibitors, we proposed a model that showed that blocking the cholesterol trafficking in endothelial cells in turn depletes membrane cholesterol in the cell membrane as well as in the lysosomal membrane. The cholesterol depletion influences membrane-bound, major angiogenic signaling proteins, such as mTOR and VEGFR2, inhibits angiogenic signaling in endothelial cells, and suppresses angiogenesis (Figure 3). Replenishing the endothelial cells with cholesterol rescued the inhibitory effect of the cholesterol trafficking inhibitors on mTOR, VEGFR2, and angiogenesis [37,49,56,57]. This observation not only validates the essentiality of the membrane’s cholesterol level in angiogenic signaling, but also proposes a hypothesis that cholesterol trafficking inhibitors may be less effective in hypercholesterolemic individuals when used for anticancer applications. This also suggests that the combination of the cholesterol trafficking inhibitors with cholesterol-lowering agents, such as HMGCR inhibitors or cholesterol absorption inhibitors, may be more beneficial for cancer treatment.

In addition to VEGFR2 and mTOR, there are a number of membrane proteins that participate in angiogenic signaling. It cannot be ruled out that cholesterol depletion may also influence other signaling molecules on the membrane in endothelial cells. Further studies are required to clarify whether certain specific proteins, or all of the membrane proteins generally, are modulated in their functions upon cholesterol depletion.

In summary, we, and other groups, have proposed that cholesterol trafficking inhibitors are potential anti-angiogenic agents based on their strong pharmacological activities. The cholesterol trafficking inhibitors introduced in this review are FDA (or foreign counterparts)-approved drugs that are well-tolerated in humans. Therefore, a repurposing of the drugs for angiogenesis or cancer treatment is actively ongoing. This study also supports the idea that endothelial cell cholesterol trafficking is a viable drug target for angiogenesis, thus providing a new target for screening and future drug discovery, and development for cancer treatment.

## Abbreviations

ABC	adenosine triphosphate-binding cassette
AIBP	apoA-I binding protein
ER	endoplasmic reticulum
HDL	high-density lipoprotein
HMGCR	3-hydroxy-3-methylglutaryl-CoA reductase
HSMC	human vascular smooth muscle cell
HUVEC	human umbilical vein endothelial cells
LAL	lysosomal acid lipase
LDL	low-density lipoprotein
LXR	liver X receptor
NPC	Niemann-Pick type C
NPC1L1	NPC1-like 1
NTD	N-terminal domain
ORP	OSBP-related protein
OSBP	oxysterol binding protein
SCAP	SREBP cleavage-activating protein
SERM	selective estrogen receptor modulators
SREBP	sterol regulatory element-binding protein
SSD	sterol-sensing domain
